# Unraveling the Metabolic Potential of Asgardarchaeota in a Sediment from the Mediterranean Hydrocarbon-Contaminated Water Basin Mar Piccolo (Taranto, Italy)

**DOI:** 10.3390/microorganisms9040859

**Published:** 2021-04-16

**Authors:** Andrea Firrincieli, Andrea Negroni, Giulio Zanaroli, Martina Cappelletti

**Affiliations:** 1 Molecular and Applied Microbiology Lab, Department of Pharmacy and Biotechnology, University of Bologna, via Irnerio 42, 40126 Bologna, Italy; martina.cappelletti2@unibo.it; 2 Department of Civil, Chemical, Environmental and Materials Engineering, University of Bologna, via Terracini 28, 40131 Bologna, Italy; andrea.negroni4@unibo.it (A.N.); giulio.zanaroli@unibo.it (G.Z.)

**Keywords:** Asgardarchaeota, Mar Piccolo, aromatic hydrocarbons, aliphatic hydrocarbons, *Thorarchaeota*, *Lokiarchaeota*, *Heimdallarchaeota*, bioremediation, metagenome

## Abstract

Increasing number of metagenome sequencing studies have proposed a central metabolic role of still understudied Archaeal members in natural and artificial ecosystems. However, their role in hydrocarbon cycling, particularly in the anaerobic biodegradation of aliphatic and aromatic hydrocarbons, is still mostly unknown in both marine and terrestrial environments. In this work, we focused our study on the metagenomic characterization of the archaeal community inhabiting the Mar Piccolo (Taranto, Italy, central Mediterranean) sediments heavily contaminated by petroleum hydrocarbons and polychlorinated biphenyls (PCB). Among metagenomic bins reconstructed from Mar Piccolo microbial community, we have identified members of the Asgardarchaeota superphylum that has been recently proposed to play a central role in hydrocarbon cycling in natural ecosystems under anoxic conditions. In particular, we found members affiliated with Thorarchaeota, Heimdallarchaeota, and Lokiarchaeota phyla and analyzed their genomic potential involved in central metabolism and hydrocarbon biodegradation. Metabolic prediction based on metagenomic analysis identified the malonyl-CoA and benzoyl-CoA routes as the pathways involved in aliphatic and aromatic biodegradation in these Asgardarchaeota members. This is the first study to give insight into the archaeal community functionality and connection to hydrocarbon degradation in marine sediment historically contaminated by hydrocarbons.

## 1. Introduction

During the past decades, hydrocarbon cycling in marine and terrestrial anoxic environments has been mostly attributed to nitrate- and sulfate-reducing communities of Delta and Betaproteobacteria catalyzing the oxidation of aliphatic and aromatic hydrocarbons most commonly found in marine sediments under anoxic conditions [1,2,3,4,5,6]. Only recently, metagenomic studies revealed that members of the Archaea kingdom, i.e., *Archaeoglobus*, Bathyarchaeota, and Asgardchaeota, carry the genetic potential to degrade short-chain alkanes, aliphatic and aromatic compounds under anaerobic conditions, suggesting that hydrocarbons degradation might not be restricted to Bacteria lineages [7]. Indeed, the Asgardarchaeota superphylum showed a surprisingly high diversity of pathways involved in hydrocarbons degradation, revealing that this superphylum members might be crucial contributors to the hydrocarbon cycling in anaerobic environments. In the last years, Asgard archaea have been detected in marine, lake and river sediments, as well as in soil and microbial mats [8]. The Asgardarchaea superphylum includes four different phyla, Thor-, Odin-, Loki-, and Heimdallarchaeota, and the novel branches named Hermod-, Hel-, Gerd- and Sifarchaeota [9,10,11,12,13,14]. Omics analysis revealed that Asgardarchaeota employs two main strategies for hydrocarbon degradation. In Helarchaeota reverse methanogenesis is activated to degrade short-chain alkanes, whereas, in Hermodarchaeota, Thorarchaeota, and Lokiarchaeota, hydrocarbon degradation pathways are similar to those observed sulfate-reducing hydrocarbon-degrading bacteria pathway where fumarate addition leads to degradation via benzoyl-CoA (for aromatic compounds) or malonyl-CoA for long-chain hydrocarbon) pathway [10,11].

Mar Piccolo is a central Mediterranean semi-enclosed marine basin connected to the Ionian sea, historically contaminated by hydrocarbons and heavy metals pollutions ascribed to decades of anthropogenic activity. Mar Piccolo underwent significant contaminations from oil refineries, marine traffics and steel production, resulting in massive pollution of heavy metals, polycyclic aromatic hydrocarbons (PAHs), and polychlorinated biphenyls (PCBs) [15]. Illumina sequencing analysis of Mar Piccolo sediments targeting 16S rDNA gene reported a microbial community highly specialized in the degradation of aromatic and aliphatic hydrocarbon mostly composed by well-known degraders of the Betaproteobacteriales, Deltaproteobacteria, and Chloroflexi bacterial groups [15]. Nonetheless, the archaeal community’s role in the biodegradation of aromatic and aliphatic hydrocarbons in this site is still unraveled. Here we describe the genetic potential of the Mar Piccolo resident Asgardarchaeaota community. In particular, whole metagenome sequencing showed that Thorarchaeota, Heimdallarchaeota, and Lokiarchaeota identified in Mar Piccolo sediments highly contaminated with crude oil and diesel fuel carry key genes needed for the degradation of aromatic and aliphatic hydrocarbons under anaerobic conditions. This study provides insights into functional genomics of Asgardarchaeota involved in the degradation of aliphatic and aromatic compounds in the Mar Piccolo basin.

## 2. Materials and Methods

### 2.1. Sediment Sample Collection and Storage

A surface sediment sample from Mar Piccolo (Taranto, Italy) was collected in 2019 by scuba diving at 12 m depth. Sediment was collected in 5 L HDPE jars (*n* = 4) filled up to the top and hermetically sealed to prevent air exposure, immediately shipped to the laboratory and stored at 4 °C until further processing.

### 2.2. Extraction and Analysis of Petroleum Hydrocarbons (PHCs) and Polychlorinated Biphenyls (PCBs)

Petroleum hydrocarbons (PHCs) and polychlorinated biphenyls (PCBs) were extracted using the Soxhlet method. Before extraction, sediments were centrifuged at 4000 rpm to remove water and air-dried overnight. One gram of air-dried sediment was mixed with sodium sulfate and 50 mL hexane:acetone (1:1) mixture 1 and extracted at 80 °C for 6 h. The solvent was vent dried, the residue suspended in 10 mL hexane, and the extract was purified via solid-phase extraction using flow-through ExtraBond^®^Sharlau cartridges “Fluorsil” (polar phase; amount 500 mg; volume 3 mL; granulometry 200 µm), eluted with hexane and collected in glass vials.

PHCs were quantified with a 6890N gas chromatograph coupled with a flame-ionization detector (GC-FID) and a 6890 series II automatic sampler (Agilent Technologies, Palo Alto, CA, USA), equipped with a 30 m HP-5 capillary column (0.25 mm ID; 0.25 μm film) under the analytical conditions described elsewhere [16], i.e., injection volume (splitless mode) = 1 µL; temperature of injector = 270 °C; temperature of detector = 320 °C; Carrier gas (nitrogen) constant pressure = 15 psi; temperature program of oven = initial temperature 60 °C, isothermal for 1 min, temperature rate 10 °C min^−1^, final temperature 320 °C, isothermal for 20 min. Diesel fuel (taken from a petrol station) was used to prepare seven points calibration curves (1000, 750, 500, 250, 100, 50, 10 mg L^−1^, r^2^ > 0.998). The recovery of PHCs from sediment was 85% ± 3%. The LOQ and RSD were 1 mg/L and <5%, respectively.

The qualitative and quantitative analysis of the extracted PCBs was performed with a gas chromatograph (6890N) equipped with an HP-5 capillary column (30 m by 0.25 mm; 0.25 μm film thickness), a ^63^Ni micro electron capture detector (microECD), and a 6890 series II automatic sampler (Agilent Technologies, Palo Alto, CA, USA) under the analytical conditions described elsewhere [17], i.e., injection volume (split ratio 9.5) = 1 µL; temperature of injector = 250 °C; temperature of detector = 320 °C; Carrier gas (nitrogen) flow rate = 1.5 mL min^−1^; temperature program of oven = initial temperature 60 °C, isothermal for 1 min, first temperature rate 40 °C min^−1^, final temperature 140 °C, isothermal for 2 min, second temperature rate 1.5 °C min^−1^, final temperature 185 °C, third temperature rate 4.5 °C min^−1^, final temperature 275 °C, isothermal for 5 min. Seven-point calibration curves were obtained using standard PCB mixtures, namely Aroclor 1260, Aroclor 1254, and Aroclor 1242, in hexane (10, 5, 2, 1, 0.5, 0.2, 0.1 mg L^−1^, r^2^ > 0.998) assuming co-eluting congeners to be present in equal proportions [18] and considering weight percentage of congeners as reported elsewhere [19]. The mean recovery of PCBs from sediment was 89% ± 2%. The LOQ and RSD were in the range 0.15–0.120 ppb (depending on the PCB congener/co-eluting congeners) and <5%, respectively.

Solvents used Met gas-chromatography specifications and were purchased by Merk (Milan, Italy). Neat (i.e., pure 100 ± 0.5%) PCB mixtures Aroclor 1242, Aroclor 1254, Aroclor 1260 were purchased by Ultrascientific Italia SRL (Bologna, Italy).

### 2.3. DNA Extraction

Total DNA was extracted from each sample (*n* = 4) using the MoBio PowerSoil DNA isolation kit (QIAGEN Inc., Valencia, CA, USA) according to the manufacturer’s instructions and finally diluted in 50 µL of nucleic acid-free ultra-pure water. Purified DNA was quantified using the BR dsDNA kit (Thermo Fisher Scientific, Waltham, MA, USA) on a Qubit 4.0 fluorometer (Thermo Fisher Scientific, Waltham, MA, USA). Equal amounts (ng µL^−1^) of DNA from each sample were pooled, achieving a final concentration of 24 ng µL^−1^.

### 2.4. Illumina Sequencing

Fifty ng of total DNA were prepared for sequencing using NEBNext^®^ Ultra™ II FS DNA library prep kit (New England Biolabs Japan, Tokyo, Japan). In total, 50 million fragments, having an average length of 300 bp, were sequenced in paired-end (PE) mode with a read length of 150 bp. Whole metagenome shotgun sequencing was performed on an Illumina NextSeq 500 at the StarSEQ sequencing facility (Muniz, Germany).

### 2.5. Reconstruction of Metagenome-Assembled Genomes (MAGs) and Functional Annotation

Raw reads were processed using BBduk to remove sequencing adapters (tbo, tpe, ktrim = r, k = 23, mink = 11) and clipping low-quality end (qtrim = rl, trimq = 15, minlength = 30, entropy = 0.5) (https://sourceforge.net/projects/bbmap/ version 38.84). High-quality reads were assembled with MEGAHIT [20], and short-length contigs (<1500 bp) filtered out using “*reformat.sh*” (https://sourceforge.net/projects/bbmap/ version 38.84). The assembled contigs were binned using three different binning algorithms CONCOCT [21], MaxBin 2.0 [22], and MetaBAT 2.0 [23], and resulting *bins* were further combined into metagenomes assembled genomes (MAGs) using metaWRAP [24]. Quality assessment of MAGs was performed within CheckM [25]. MAGs open reading frames were detected using prodigal within the RAST-tk annotation pipeline implemented in the PATRIC platform [26,27,28]. Protein coding genes were submitted to eggNOG for functional annotation [29]. For the gene of interest, eggNOG functional annotation was manually curated by identifying protein domains InterProScan [30]. Carbohydrate degrading enzymes, peptidase and hydrogenase coding genes were predicted using dbCAN, MEROPS, and HydDB databases, respectively [31,32,33]. To detect membrane-bound peptidase and secreted carbohydrate-active enzymes, TMHMM v2.0 (http://www.cbs.dtu.dk/services/TMHMM/) and SignalP-5.0 were used [34].

### 2.6. Phylogenetic Analysis of Alkyl (Ass) and Benzyl-Succinate Synthase (Bss), Sulfhydrogenase, and Reductive Dehalogenase Genes

Putative Ass and Bss protein sequence were downloaded from Zhang et al. [11] and used as a reference database to query Asgardarchaeota proteome using DIAMOND [35]. Hits having a query coverage above 60% and a minimum percentage identity of 35% were selected for phylogenetic analysis. Putative Ass/Bss for the reference database and Mar Piccolo Asgardarchaeota were aligned to each other using MAFFT, and non-conserved regions were trimmed using trimAl [36,37]. Trimmed alignments were then analyzed with FastTree using the LG amino acid substitution model [38]. Asgardarchaeota putative sulfhydrogenase were aligned using MAFFT against the sulfhydrogenase complex I and II from *Pyrococcus furiosus* DSM 3638, and a phylogenetic tree was constructed in IQ-TREE applying the substitution model LG + F [39]. Protein coding sequence having the reductive dehalogenase (Rdh) domain IPR028894 were aligned using MAFFT against a custom database from Zhang et al. [11] amended with putative Asgardarchaeota Rdh proteins downloaded from UniProtKB/TrEMBL (release 2020_05). The alignment file was then analyzed with IQ-TREE, enabling the best substitution model (WAG + F + I + G4). All phylogenetic trees were computed applying an SH-like approximate likelihood ratio test with 1000 permutation [40].

### 2.7. Phylogenomic Analysis

Phylogenetic relatedness of MAGs having completeness above 50% was assessed in two steps. First, GTDB-Tk [41] was used to determine the taxonomy of MAGs/bins and a custom database, including all representative proteomes in the taxonomic lineage using GToTree [42]. Second, for each genome in the database, including the MAG of interest, the 76 archaeal phylogenetic markers were concatenated with GToTree, and a phylogenetic tree was built in FastTree with the -lg (LG + CAT model) parameter enabled.

## 3. Results and Discussion

### 3.1. Sampling Site Description

In several studies, Mar Piccolo sediments were described as heavily contaminated with heavy metals (especially mercury) at 9 mg/kg sediment, polycyclic aromatic hydrocarbons up to 12.7 mg/kg sediment, and PCBs up to 1.7 mg/kg sediment [15,43,44]. The sediment collected in this study showed a high concentration of PHCs: 1.38 ± 0.10 g/kg of sediment (dry weight). PCB contamination was assessed, too, revealing a total concentration of 10.6 ± 0.05 mg/kg of sediment (dry weight) (Table 1).

The relative concentrations of the contaminants were expected to select for microbial community specialized in the degradation of aliphatic and aromatic hydrocarbons rather than PCBs, the latter being present at concentrations two orders of magnitude lower and being notably more recalcitrant to biodegradation than PHCs under anaerobic conditions.

### 3.2. Assembly Results and Phylogenomic Analysis of Asgardarchaeota MAGs from the Mar Piccolo Sediment

**Figure 1:**
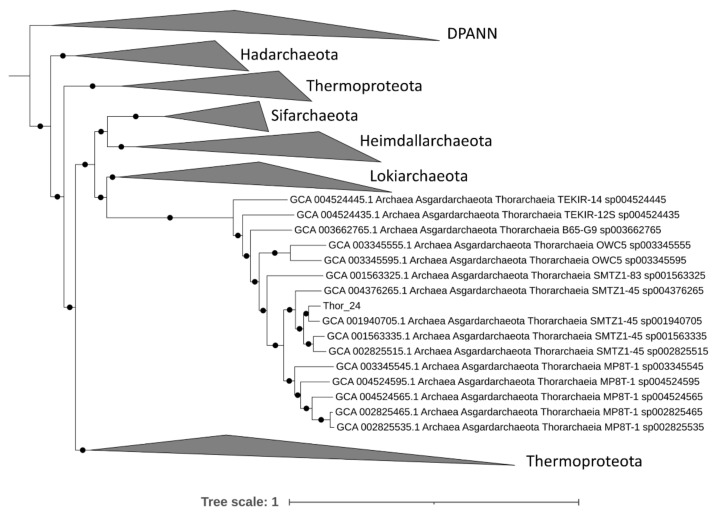
Phylogenomic analysis of Thorarchaeota metagenome-assembled genome (MAG) (Thor_24) based on 76 archaeal phylogenetic markers using IQTree. Only nodes supporting a bootstrap value above 0.7 are shown. Diapherotrites, Parvarchaeota, Aenigmarchaeota, Nanoarchaeota and Nanohaloarchaeota (DPAAN) was manually placed as an outgroup. Taxonomy nomenclature from the GTDK database is displayed as well as the GenBank Accession numbers.

A total of 34,682 contigs having a minimum length of 1500 bp and an average GC content of 47% were assembled from approximately 47 million high PE reads. Only 17% of the assembled high-quality reads mapped against contigs, suggesting that a large fraction of reads were not assembled or assembled in contigs with a length lower than 1500 bp. Among these, metaWRAP retrieved 1 high-quality MAG (completeness > 90% and contamination < 5%), 9 medium-quality MAGs (completeness > 50% and contamination < 10%) and 17 low-quality MAGs (completeness < 50%) (Table S1). The fact that only a limited number of high/medium-quality MAGs was retrieved indicates that the Mar Piccolo sediment resident microbial community has a remarkable sequence diversity; hence the assembly graph was hardly resolvable in longer contigs, possibly due to a very high intraspecific diversity. Most of the archaea bins grouped in the phylum of Asgardarchaeota, while others in Crenarchaeota and Euryarchaeota (Table S1). The phylogenomic analysis performed on 76 archaeal phylogenetic markers confirms the affiliation of the Thor_24 to the Thorarchaeota phylum (Figure 1), as Thorarchaeota AB-25 was the closest genome to this MAG.

Accordingly, Thor_24 shared an average nucleotide identity (ANI) of 80% and an average amino acid identity (AAI) of 75% with AB-25, indicating that both Asgardarchaeota belongs to the same genus (Table S1). The GTDB-Tk phylogenetic analysis of low-quality Asgardarchaeota bins was attempted despite the low completeness. These bins (i.e., loki_1, loki_4 loki_6, and heim_22) resulted in being affiliated with Lokiarchaeota and Heimdallarchetoa, respectively. However, the low AAI (Table S1) suggests that Loki and Heim bins could belong to phylogenetically distinct lineages within the Lokiarchaeota and Heimdallarchaeota phyla.

### 3.3. Genetic Potential of Asgardarchaeota Mar Piccolo Resident Microbial Community

#### 3.3.1. Carbohydrate and Peptides Degrading Enzymes

The capability of carbohydrate degradation was investigated by assigning protein-coding genes to families of carbohydrate-active enzymes (CAZy). Only a limited number of CAZy families was found in Thor_24, which is in agreement with other Thorarchaeaota identified in river estuary (SMTZ) and coastal sediments (AB) [45,46]. In addition, none of the CAZy families identified in Thor_24 possessed a signal peptide, suggesting that these enzymes are not actively involved in the extracellular degradation of carbohydrates. The lack of secreted CAZy enzymes does not necessarily prevent Thorarchaeota to ferment organic carbon. Indeed, among the CAZy found in Thorarchaeota, the families shared by members of this taxonomic group were GH109, GH38, and GH36, which include enzymes with a hydrolytic activity towards N-acetylgalactosamine, α-mannosides, and α-galactosides, respectively (Table S2). Therefore, these enzymes may enable members of this taxonomic group to scavenge readily degraded carbohydrates derived from the hydrolytic activity of other microorganisms. Despite the low completeness, Loki and Heim bins carried a more diversified set of CAZy families. α-N-acetylgalactosaminidase (GH109), α-mannosidases (GH38) and α-galactosides (GH36) were also found in Loki bins in addition to α-amylase (GH57), β-galactosidase/mannosidase/glucuronidase (GH2) and CE14 (chitooligosaccharide deacetylases). CAZy enzymes identified in Loki bins did not show a signal peptide domain. Finally, heim_bin carried a higher number of carbohydrate esterases (CE) families, including the CE14, also detected in Loki bins, and CE1, CE11, and CE4, which comprise enzymes catalytically active towards phenolic polymers (e.g., lignin), and lipopolysaccharides (Table S2). With such a diversified set of CAZy families, heim_22 shows a stronger degradative potential towards carbohydrates than Thor_24 and Loki bins.

In addition to CAZy enzymes, the capacity to degrade peptides and oligopeptides was investigated by assessing the presence of membrane-bound peptidase and amino acid/oligopeptide transport systems. Asgardarchaeota MAG contains multiple membrane-bound and soluble peptidases (M, metallopeptidase; S, serine peptidase, A, aspartic peptidase) together with genes coding for components of the oligo- and dipeptide transporters (Opp and Dpp) and branched amino acid transport system Liv (Table S3). The presence of genes encoding enzymes involved in the breakdown of poly- and oligo-peptides together with Opp, Dpp and Liv systems, suggests that these microorganisms could utilize amino acids, oligopeptides and proteins as both carbon and nitrogen sources. In conclusion, the presence of CAZy enzymes and peptidase is consistent with the proposed heterotrophic lifestyle of Asgardarchaeota [8,45,47].

#### 3.3.2. Central Metabolism

**Figure 2:**
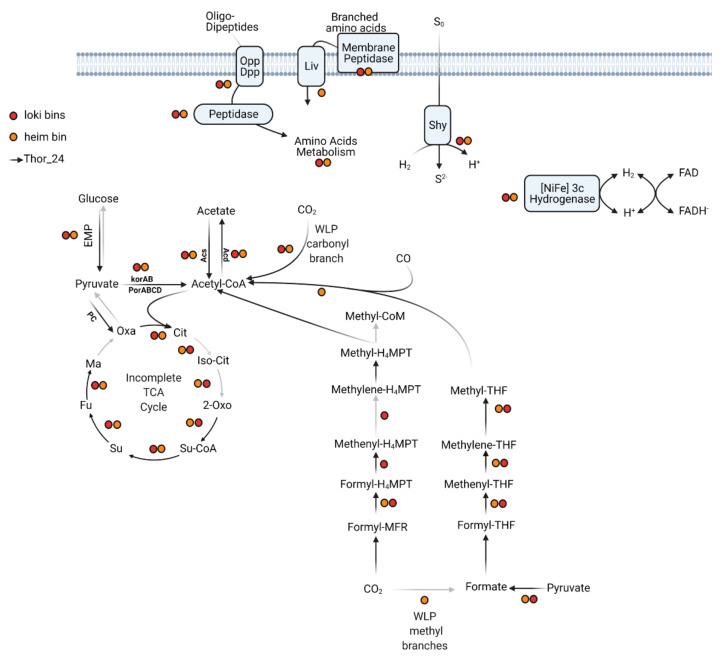
Central metabolism of Thorarchaeota MAG (Thor_24) and Loki- (Loki) and Heimdall- (Heim) archaeota low-quality bins. Gray arrows indicate pathways/reactions missing in Thor_24. Colored dots indicate the presence of enzymes in low-quality bins (red for Loki and orange for Heim). The annotation files for each pathway are reported in Tables S2 and S3.

The Wood–Ljungdahl pathway (WLP) includes a series of enzymes catalyzing the reduction of two molecules of CO_2_ into acetyl-CoA through two different pathways, i.e., the methyl and carbonyl branches [48]. Depending on the C_1_ carrier, two variants of the WLP have been characterized so far. Concerning the C_1_ carriers, the acetogenic bacteria use tetrahydrofolate (THF), whereas the methanogenic Archaea, which couple methanogenesis to CO_2_ reduction, utilize the tetrahydromethanopterin (THMPT) as carrier [49]. While the carbonyl-branch can be found in both Bacteria and Archaea, the THF-WLP and THMPT-WLP paths of the methyl branch are, respectively, found in acetogenic bacteria and methanogenic Archaea. Like in other Thorarchaeota, the genes involved in the reduction of CO_2_ into acetyl-CoA through the WLP methyl and carbonyl branches were detected in Thor_24 (Figure 2). Coding genes of the subunits of the enzymatic complex CODH/ACS catalyzing the reduction of CO2 through the carbonyl branch were found in Thor_24 and Thorarchaeota MAGs AB and SMTZ (Figure 2) [45].

Enzymes of the WLP pathway carbonyl branch were also detected in Loki and Heim bins. Both pathways of the methyl branch require the multiple-stage reduction of CO_2_ through the formation of formate/formyl, methenyl, methylene, and finally, a methyl group. The genes catalyzing each reduction step were detected in Thor_24 MAG except for the enzymes of the formate dehydrogenase complex, which catalyzes the conversion of CO_2_ into formate within the WLP-THF branch. However, Thor_24 carried multiple copies of the pyruvate-formate lyase (pfl), indicating that formate, rather than CO_2_, is the main substrate of the enzymes of the WLP-THF branch (Table S3). Therefore, Thor_24 potentially performs autotrophic carbon fixation through the WLP methyl branch THMP. Partial sets of WLP-THMP methyl branch enzymes were also detected in Loki and Heim bins (Figure 2), possibly due to the poor completeness of the respective bins. Indeed, Lokiarchaeota were shown to possess a complete WLP, comparable to that observed in Thorarchaeota [8]. On the other hand, the lack of WLP enzymes seems to be common in Heimdallarchaeota [50,51]. Furthermore, the presence of enzymes catalyzing the conversion of acetyl-CoA into acetate via acetyl-CoA synthetase (acd, ADP-forming) indicates that Thor_24 possesses the potential to synthesize reducing equivalents in the form of acetate via CO_2_ fixation or fermentation of organic substrates (Figure 2). Acd coding genes were also detected in the Heim bin. Finally, Several components of the tricarboxylic acid (TCA) cycle were missing in Thor_24 as well as in Loki and Heim bins. The missing enzymes in Thor_24 were the aconitate hydratase, isocitrate dehydrogenase, and malate dehydrogenase, catalyzing, respectively, the second, third and last reactions of the TCA cycle (Figure 2).

#### 3.3.3. Sulfur and Nitrogen Metabolism

**Figure 3:**
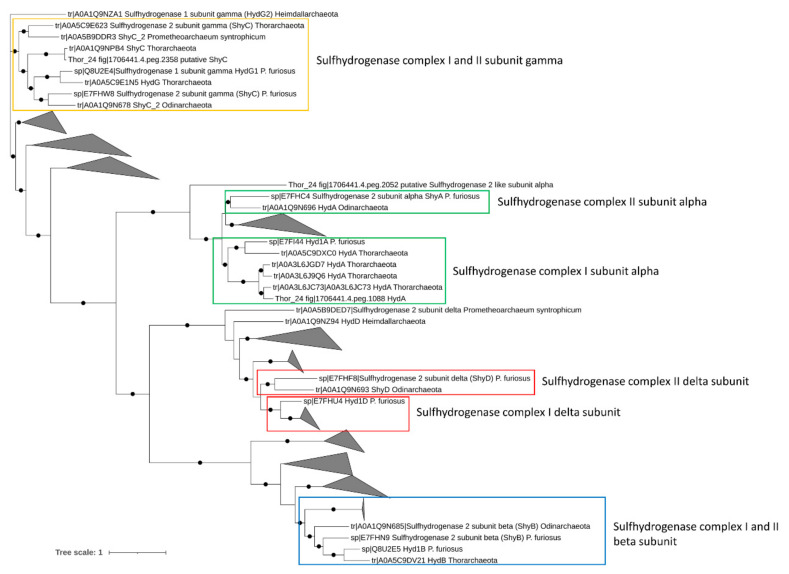
Phylogenetic three of sulfhydrogenase complex I and II genes in Mar Piccolo Asgardarchaeota. Only nodes supporting a bootstrap value above 0.7 are shown. GenBank Accession numbers and Uniprot accession numbers are shown.

Energetically favorable oxidation of hydrocarbons in anaerobic environments is coupled to the utilization of a final electron acceptor such as nitrate and sulfate [10]. Therefore, the presence of genes involved in nitrate and sulfate reduction was assessed in Asgardarchaeota bins. While multiple nitrite-reductase coding genes (nir) catalyzing the dissimilatory reduction of nitrite to ammonia were found in Thor_24, none of these genes was organized in a cluster with other genes of the Nir complex. This suggests the absence of terminal reductases involved in nitrite/nitrate reduction in Thor_24. Anaerobic sulfite reductase (asr) genes and dissimilatory sulfite-reductase (dsr) genes were also missing in the Thor_24 as well as in the Asgard archaea bins. Lack of *dsr* and *asr* genes in Asgardarchaeota was previously reported suggesting that these organisms do not perform dissimilatory sulfate reduction [8]. On the contrary, members of the [NiFe] group-3b hydrogenase have been proposed to possess a sulfhydrogenase activity, catalyzing the reduction of elemental sulfur S(0) and polysulfides into hydrogen sulfide, using H_2_ as electron donor [52]. *Pyrococcus furiosus* sulfhydrogenase complex, I (Hyd) and II (Shy), are two cytoplasmatic enzymes possessing different catalytic activity and affinity towards S(0) and polysulfide. As compared to complex Hyd, the Shy complex II showed higher affinity towards S(0), and therefore, becoming relevant when sulfur levels are lows [53]. Additionally, in the absence of S(0) or polysulfides, Shy complex II can work in reverse utilizing not NADH as electron donors for H_2_ generation [53]. While Thor_24, Loki, and Heim bins carried one copy of the catalytic subunit of NiFe group-3b hydrogenase, only Thor_24 HydA clusters with the sulfhydrogenase 1 subunit alpha (Hyd1A) of *P. furiosus*, which possess a sulfur-reducing activity under in vitro condition (Figure 3) [54].

Evidence of sulfhydrogenase genes has been found in Thorarchaeota MAGs recovered from river estuary, suggesting that Thorarchaeota could potentially perform hydrocarbon degradation while using sulfur as electron sinks [14]. Finally, in addition to sulfhydrogenase I complex genes, Thor_24 and heim_bin carried two clusters encoding for the subunits A and B of the electron transfer flavoproteins EFTAB (Table S3), which can transfer electrons from succinate conjugates to a pool of quinone during the degradation of aromatic and aliphatic hydrocarbons [55].

#### 3.3.4. Identification of Syntrophic Lifestyle Genes in Throarchaeota, Lokiarchaeota, and Heimdalarchaeota Bins

Redox-active complex, such as NADH-quinone and NADH-ubiquinone oxidoreductase and external cytochromes, could potentially take part in syntrophic interaction with a sulfate/nitrate-reducing bacterium. Such interactions have been hypothesized for members of the Asgardarchaeota superphylum, such as Hermodarchaeota, Heimdallarchaeota, Helarchaeota, and Lokiarchaeota [10,47,52]. Additionally, several MAG identified in this work were affiliated with lineages of sulfate-reducing bacteria (Table S1). Hence, Thor_24 and low-quality bins were screened for genetic potential associated with interspecies interactions, such as flagellin and pili coding genes. We found that one member of the loki_bins (loki_4) harbors a gene encoding an archaeal type flagellin protein (IPR002774), while Thor_24 carries multiple copies of quinone and ubiquinone oxidoreductase, which could act as energy transferring complex in syntrophic interactions (Table S3). However, the lack of extracellular cytochrome, which mediates electron transfer across syntrophic partners, poses some questions about the capability of Thor_24 and loki_4 to engage in syntrophic interactions with sulfate-reducing partners inhabiting Mar Piccolo identified in Quero et al. [15] and in this study (Table S1).

#### 3.3.5. Analysis of Genes Involved in Petroleum Hydrocarbon Degradation and Reductive Dehalogenation

**Figure 4:**
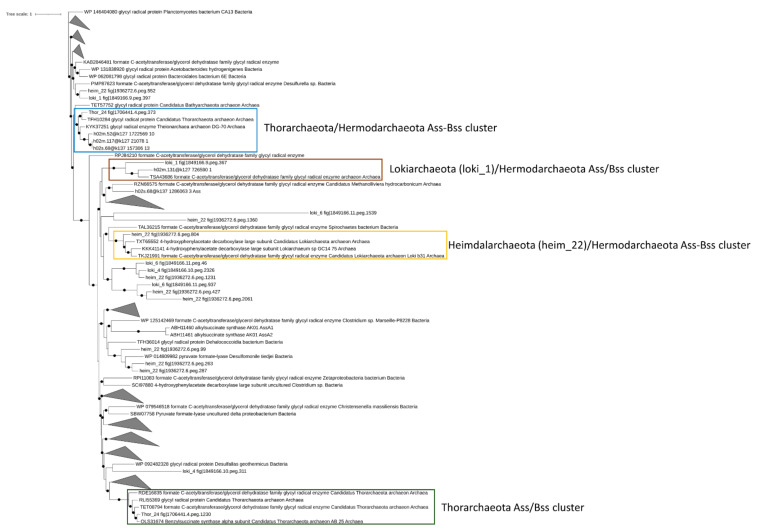
Phylogenetic tree of putative benzyl-alkyl-succinate synthase in Mar Piccolo Asgardarchaeota. Only nodes supporting a bootstrap value above 0.7 are shown. GenBank Accession numbers and Uniprot accession numbers are shown.

Under anaerobic conditions, the degradation of aromatic and aliphatic hydrocarbons was attributed to the catalytic activity of benzyl/alkyl succinate synthase (Bss and Ass) and methyl-CoM reductase (mcr), respectively [56,57]. Based on previous studies, Bss/Ass proteins catalyze the conversion of aryl/alkyl hydrocarbons into the corresponding succinate conjugates through the fumarate addition to the benzylic carbon, in the case of aromatic compounds, or to the terminal/subterminal carbon of n-alkanes [57,58]. The degradation of short-chain alkanes, i.e., ethane, propane, and butane, under anaerobic conditions has been associated with the *mcr* gene products, which catalyze the alkane oxidation to acetyl-CoA through a reversal of the last step in methanogenesis [59]. While in marine sediments, the degradation of aryl and alkyl-hydrocarbons were mostly attributed to sulfate-reducing bacteria, recently, the ecological role of Asgardarcheota has been proposed in hydrocarbon cycling. Therefore, we sought to determine the presence of aryl and alkyl-hydrocarbons degrading enzymes in Asgardarcheota detected in Mar Piccolo sediments. Putative Bss/Ass proteins were detected in Thor_24 and, Lokiarchaeota and Heimdallarchaeota bins (Figure 4). However, only Thor_24, loki_1, and heim_22 Bss/Ass protein clustered with benzyl and alky-succinate synthase previously identified in Thorarchaeota, Lokiarchaeota and Hermodarchaeota [11](Figure 4).

**Figure 5:**
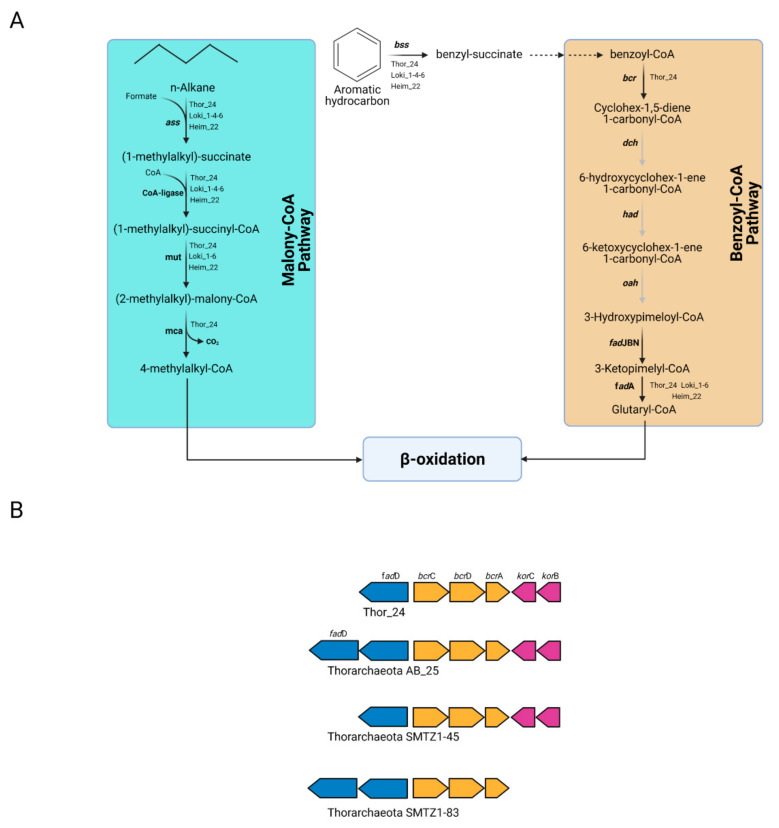
Metabolic pathways in Thorarchaeota, Lokiarchaeota, and Heimdallarchaeota bins involved in the degradation of aliphatic and aromatic hydrocarbons. (**A**) Malonyl-CoA and benzoyl-CoA pathways predicted in Thor_24 (Th24), Loki (Lk) and Heim (Hm) bins; gray arrows indicate that the reaction is not supported by the presence of the enzyme in the genome; genes involved in the pathways shown in this figure are reported in Table S3. (**B**) Gene cluster of the benzoyl-CoA-reductase genes (bcr) in Thor_24 and Thorarchaeota AB_25, SMTZ1-45, and SMTZ1-83. The Integrated Microbial Genome database was used identify bcr gene neighborhood of AB_25 (locus tag Ga197643_102918-102921), SMTZ1-45 (Ga0197632_115520-115514), and SMTZ1-83 (Ga0197634_10836-10831).

Therefore, the capability to degrade aromatic and aliphatic compounds may not be restricted to bacterial taxa previously identified in Mar Piccolo sediment [15]. Once the substrate is activated, the succinate conjugates deriving from aromatic and aliphatic hydrocarbons can be further oxidized through the benzoyl-CoA (bCoA) and methylmalonyl-CoA (mmCoA) pathways. The first reaction step of the bCoA pathway involves the benzoyl-CoA reductase activity (encoded by *bcr* gene), which catalyzes the conversion of benzoyl-CoA into cyclohexa-1,5-dienecarbonyl-CoA. Genes coding for the subunits of the Bcr complex were found in the sequence of Thor_24 (Figure 5A).

In particular, Thor_24 bcr genes showed the same organization observed in Thorarchaeota MAGs AB and SMTZ, whereas this gene arrangement could not be detected in Loki and Heim bins due to their fragmented genome (Figure 5B). Compared to Hermodarchaeota, most of the genes of the bCoA pathway were missing in Thor_24, but also in Loki and Heim bins. For instance, the *dch* (cyclohexa-1,5-dienecarbonyl-CoA hydratase) *had* (6-hydroxycyclohex-1-ene-1-carbonyl-CoA dehydrogenase) and *oah* (6-oxocyclohex-1-ene-carbonyl-CoA hydrolase) genes, which are involved in the synthesis of hydroxy-pimeloyl-CoA from benzoyl-CoA, were missing (Figure 5A), were not detected in Mar Piccolo Asgardarchaeota. Therefore, despite the presence of the *bcr* genes, Thor_24 unlikely possesses the capacity to degrade aromatic hydrocarbons through the bCoA and finally into the β-oxidation pathway. Conversely, the degradation of aromatic hydrocarbons in Thorarchaeota may occur through an alternative and yet undiscovered pathway. While the anaerobic degradation of aryl-succinates through the bCoA pathway is well characterized, the mmCoA pathway has been recently proposed as a candidate route for the degradation of alky-succinate conjugates [3]. MmCoA pathway enzymes catalyzing the epimerization (MUT), decarboxylation (MCA), and subsequent assimilation through the β-oxidation pathway have been identified in Thor_24 and Loki and Heim archaea bins (Figure 5A). Therefore, hydrocarbon cycling carried out by Asgardarcheota in Mar Piccolo preferentially involves the utilization of alkyl degrading enzymes over the aromatic ones. Finally, along with the Ass/Bss proteins, Asgardarcheota were proposed to catalyze the conversion of alkane to alkyl-CoM through the catalytic action of a methyl-CoM-reductase-like enzyme [10]. Alkyl-CoM is further reduced to acyl-CoA through the action of the heterodisulfide reductase encoded by hdr genes, which are needed for the recovery of the CoM. While archaeal hdr genes were found in Thor_24 and low-quality Asgardarcheota bins, the genes coding for putative Mcr enzyme were missing in these genomes. In conclusion, these results suggest that alkane degradation in Mar Piccolo Asgardarchaeota is catalyzed by the ass-like genes.

**Figure 6:**
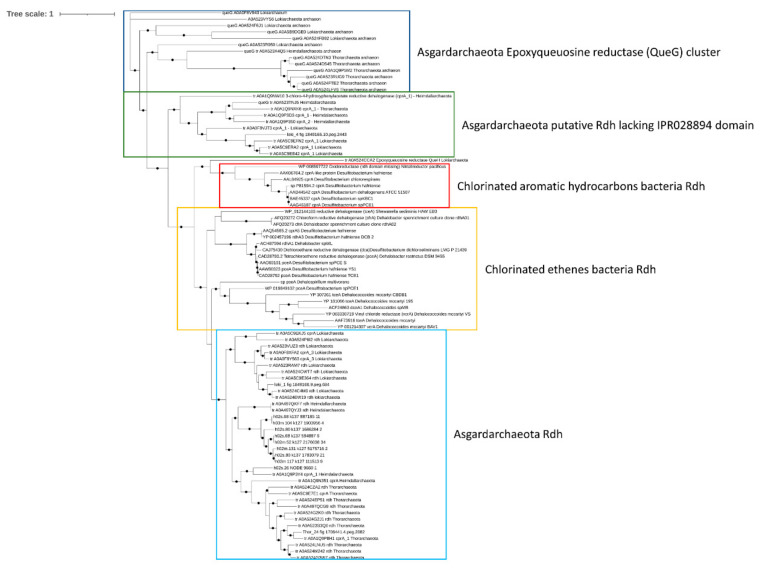
Phylogenetic tree of putative reductive dehalogenase (Rdh) in Thor_24, Loki and Heim bins, and Asgardarchaeota. Only nodes supporting a bootstrap value above 0.7 are shown. GenBank accession numbers and Uniprot accession numbers are shown. Epoxyqueuosine reductases (QueG) from Asgardarchaeota were used as an outgroup.

Reductive dehalogenase (rdh) genes in organohalide-respiring bacteria have been linked to the anaerobic dehalogenation of PCBs and other halogenated hydrocarbons in marine and terrestrial environments [60]. While reductive dechlorination via *rdh* genes has been well studied in anaerobic bacteria, the ecological role of archaea, and in particular Asgardarcheota, in the degradation of halogenated hydrocarbons and PCBs has been only hypothesized. Specifically, rdh genes have been identified in almost every phylum of the Asgardarchaeota superphylum [10,11]. Therefore, the presence of *rdh* genes in Mar Piccolo Asgardarchaeota was assessed. The enzymes linking the dechlorination with cellular respiration have been identified only in the Thor_24 and loki_1 bin (Figure 6).

Phylogenetic analysis confirmed the affiliation of these proteins to putative Rdh previously characterized in Asgardarchaeota, clustering together with known bacterial Rdh showing catalytic activity towards chlorinated ethenes [11,60]. Interestingly, the Rdh from loki_4 was included in a cluster of Asgardarchaeota-reductive dehalogenase lacking the dehalogenase domains (IPR028894). Although the RdhA from Asgardarcheota shares the same functional domain IPR028894 with Rdh proteins of bacterial origin, they lack the signal peptide; therefore, these enzymes’ actual involvement in the reductive dechlorination is still an open question [61].

## 4. Conclusions

In this study, the metabolic potential of Asgardarcheota inhabiting sediment from the Mar Piccolo basin was analyzed to dissect the genetic determinants involved in the degradation of aromatic and aliphatic compounds in this contaminated marine basin. New taxa in the branch of the Asgardarcheota group has been previously proposed to be involved in hydrocarbon cycling, expanding previous studies mostly reporting sulfate and nitrate/nitrite reducing bacteria as main degraders. We found that the Asgardarcheota phyla Thorarchaeaota, Lokiarchaeota, and Heimdallarchaeota inhabiting the Mar Piccolo sediment under analysis in this study possess the genetic potential to degrade hydrocarbons via the formation of alky-succinate conjugates, which are further degraded through the mmCoA pathway. These features suggest that the archaeal community associated with this Mar Piccolo sediment is, as a whole, a contributor to hydrocarbon cycling in this environment. Taken together, our results expand the knowledge on the contribution of Asgardarcheota to hydrocarbon cycling biodegradation in highly contaminated marine environments, pointing out that further research is needed to unravel the extensive ecological role of members of this superphylum that is still underestimated.

## Figures and Tables

**Table 1:** Concentration of petroleum hydrocarbons (PHCs) and polychlorinated biphenyls (PCBs) in the Mar Piccolo sediment.

Pollutant	Concentration
PHCs (g/kg_dw_)	1.38 ± 0.10
Di-chlorobiphenyls (mg/kg_dw_)	3.36 ± 0.02
Tri-chlorobiphenyls (mg/kg_dw_)	1.05 ± 0.00
Tetra-chlorobiphenyls (mg/kg_dw_)	0.98 ± 0.00
Penta-chlorobiphenyls (mg/kg_dw_)	0.83 ± 0.00
Hexa-chlorobiphenyls (mg/kg_dw_)	2.26 ± 0.01
Hepta-chlorobiphenyls (mg/kg_dw_)	1.07 ± 0.41
Octa-chlorobiphenyls (mg/kg_dw_)	0.35 ± 0.00
Total PCBs (mg/kg_dw_)	10.6 ± 0.05

## Data Availability

Genome sequence data are available in the NCBI database under BioProject ID PRJNA708323. Thor_24 whole-genome sequence was deposited at DDBJ/ENA/GenBank under the accession JAFVIN000000000. Genome annotation files (general feature format (.gff) and protein fasta (.faa)) described in this paper are available at https://osf.io/pwy6u/ (accessed on 14 March 2021).

## Supplementary Materials

The following are available online at https://www.mdpi.com/article/10.3390/microorganisms9040859/s1, Table S1: Metawrap bin stats and taxonomic classification via GTDB-tk; Table S2: Peptidase and Carbohydrate active enzymes identified in Mar Piccolo Asgardarchaeota; Table S3: Functional annotation via eggNOG.
